# Selecting Goals and Target Muscles for Botulinum Toxin A Injection Using the Goal Oriented Facilitated Approach to Spasticity Treatment (GO-FAST) Tool

**DOI:** 10.3390/toxins15120676

**Published:** 2023-11-29

**Authors:** Jorge Jacinto, Alexander Balbert, Djamel Bensmail, Stefano Carda, Nathalie Draulans, Thierry Deltombe, Nicholas Ketchum, Franco Molteni, Rajiv Reebye

**Affiliations:** 1 Alcoitão Medical Rehabilitation Centre, Rua Conde Barão, 2649-506 Alcabideche, Portugal; 2 Department of Adaptive Physical Training, Ural University of Physical Education, Sverdlovsk Regional Hospital for War Veterans, 620014 Yekaterinburg, Russia; 3 Department of Physical and Rehabilitation Medicine, Raymond-Poincaré Teaching Hospital, APHP, Université Paris-Saclay, 92380 Garches, France; 4 Unité INSERM 1179, University of Versailles Saint-Quentin-en-Yvelines, 78180 Montigny-Le-Bretonneux, France; 5 Department of Clinical Neurosciences, Service of Neuropsychology and Neurorehabilitation, Lausanne University Hospital (CHUV), 1011 Lausanne, Switzerland; 6 Department of Rehabilitation, Libra Rehabilitation and Audiology, 5022 KE Tilburg, The Netherlands; 7 Department of Physical Medicine and Rehabilitation, Université Catholique de Louvain, Centre Hospitalier Universitaire de Namur, Godinne Site, Avenue Docteur G Therasse, 5530 Yvoir, Belgium; 8 Department of Physical Medicine and Rehabilitation, Medical College of Wisconsin, 9200 W., Milwaukee, WI 53226, USA; nketchum@mcw.edu; 9 Villa Beretta Rehabilitation Center, Valduce Hospital, 23845 Costa Masnaga, Italy; franco56.molteni@gmail.com; 10 Department of Medicine, Division of Physical Medicine and Rehabilitation, University of British Columbia, Vancouver, BC V5Z 2G9, Canada

**Keywords:** GAS, goal-setting, GO-FAST, SMART treatment goals

## Abstract

The objective of this article is to introduce the GO-FAST Tool (developed by the Toxnet group) to clinicians working in the field of neurological rehabilitation, specifically post-stroke spasticity management. The concepts utilized in the Tool and described in this article can be broadly grouped into five topics: the principles of patient-centred goal-setting; an algorithm for setting SMART (specific, measurable, attainable, realistic, and timed) treatment goals; goal-related target muscles and botulinum toxin type A dose determinants; goal attainment follow-up, scoring, and interpretation; and the multimodal approach to spasticity management. The Tool can enhance clinical practice by providing guided assistance with goal-setting and target muscle selection for botulinum toxin type A treatment. It also provides support with the follow-up evaluation of goal attainment and calculation of treatment success. The Tool is designed to be used by clinicians with varying levels of expertise in the field of neurological rehabilitation and post-stroke spasticity management, from those who are new to the field to those with many years of experience. A case study is presented in the Results Section of the article to illustrate the utility of the Tool in setting SMART treatment goals in the management of patients with post-stroke spasticity.

## 1. Introduction

Spasticity is common after stroke and is a major cause of stroke-related disability and pain [1,2,3,4]. Therefore, the management of spasticity is crucial in post-stroke care. The injection of botulinum toxin A (BoNT-A), a protein neurotoxin derived from Clostridium botulinum, is one of the most effective and well-established therapies for post-stroke spasticity [5,6,7]. The management of spasticity is challenging due to the diversity of patient presentations, functional status, expectations, and treatment goals [1]. Patient-centric goal setting is, therefore, an essential part of the multidisciplinary therapeutic process when treating patients with spasticity [1,8,9].

**Figure 1:**
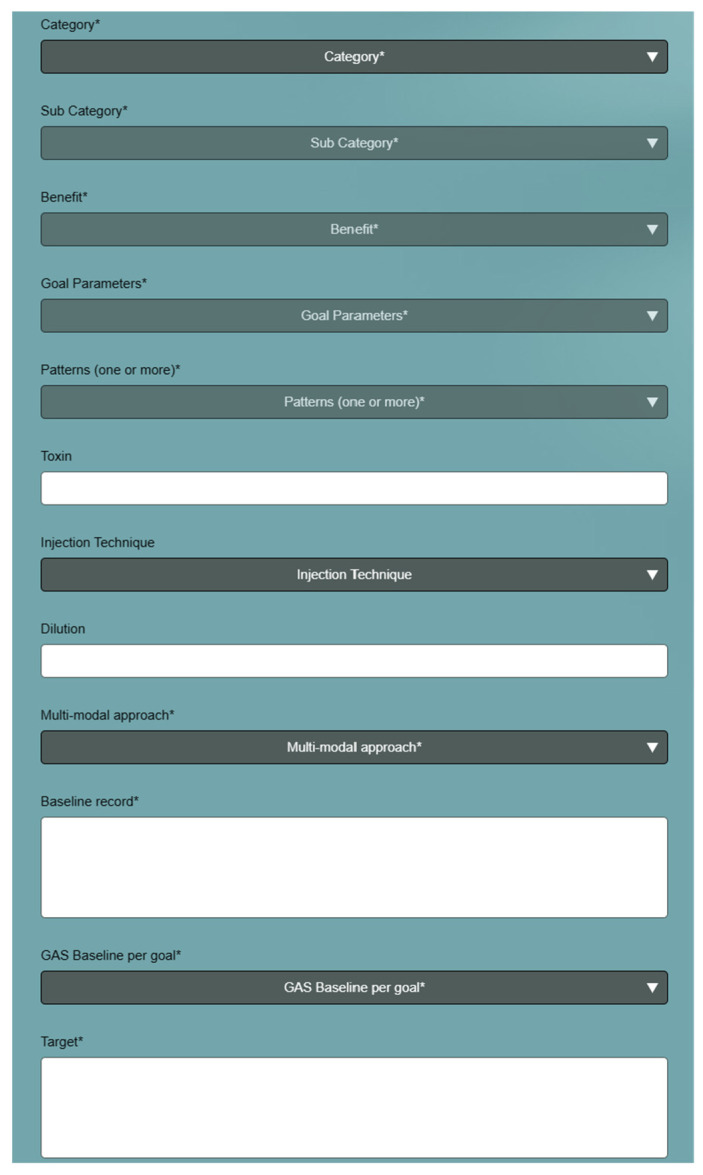
Screenshot of the GO-FAST Tool. * designates mandatory inputs.

Toxnet is an international group of expert physicians specializing in spasticity management that aims to facilitate improvements in education and provide guidance to deliver equitable and optimal spasticity management worldwide. This group identified opportunities to improve education and optimize spasticity care by developing and disseminating a comprehensive educational curriculum resource [10]. The specific need for a resource to assist in goal setting and selection of target muscles for BoNT-A injection in patients with post-stroke spasticity was identified following discussions between clinicians and academics working in the field of neurorehabilitation. The GO-FAST (Goal Oriented Facilitated Approach to Spasticity Treatment) Tool (https://go-fast.toxnet.net, accessed on 27 November 2023) was developed as such a resource (see Figure 1).

## 2. Results

### 2.1. GO-FAST Case Study to Demonstrate the Applicability of the Tool 

A hypothetical case study is presented to demonstrate the use of the GO-FAST Tool and to illustrate the principles of setting and monitoring SMART treatment goals. 

The hypothetical case is a 52-year-old woman with left hemiparesis following ischemic stroke. She presents to the clinic with upper- and lower-limb spasticity and is accompanied by her caregiver. She struggles to place her hand splint, and the caregiver reports issues with performing hand hygiene on the patient. The patient also has trouble sleeping and enduring mobilizations due to shoulder pain. Given that the three problems raised are related to spasticity, they can be considered goals for multimodal treatment, including BoNT-A.

**Figure 2:**
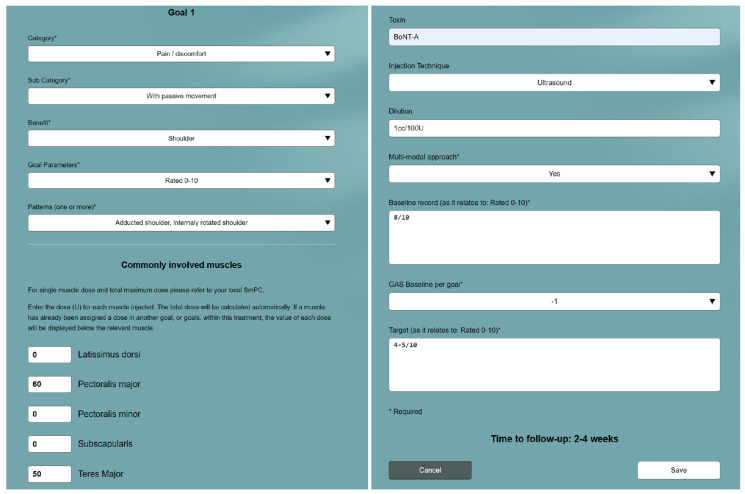
Using the GO-FAST Tool to set the primary goal in the case study.

**Figure 3:**
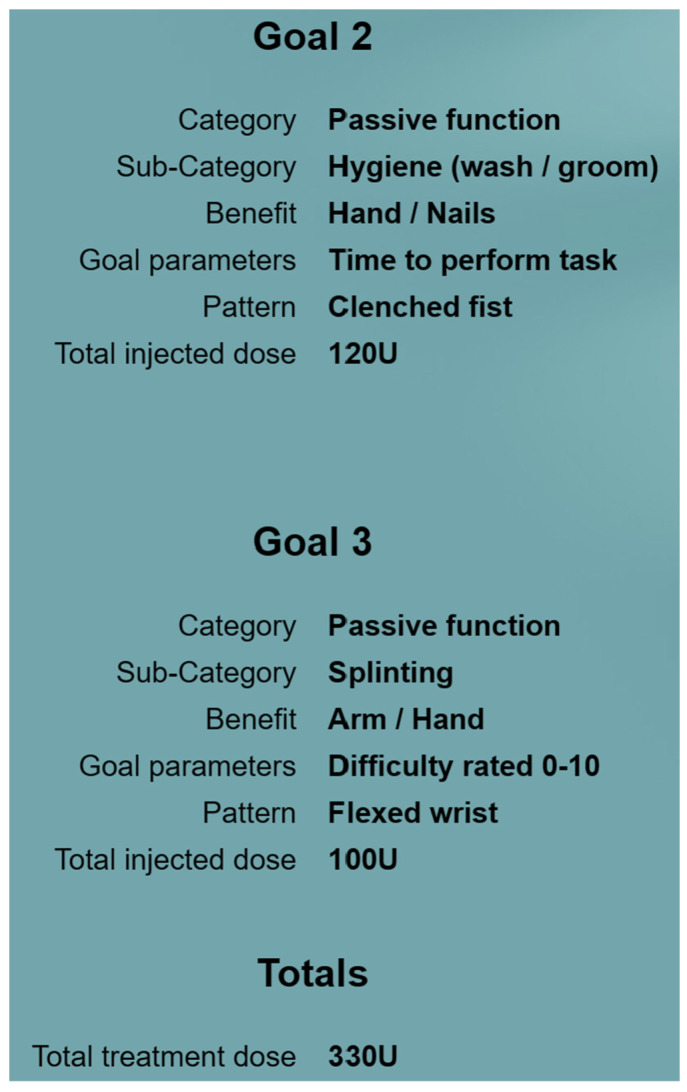
Using the GO-FAST Tool to set the remaining goals in the case study.

The clinical assessment identifies three goal-related spastic muscle patterns to be targeted according to patient and caregiver priorities. To increase patient and caregiver engagement with the treatment plan and the likelihood of achieving the goal, a mixture of short and long-term goals may be discussed. Once there is consensus between the patient, caregiver, and clinician on how achievable and realistic the goals are, a decision is made to treat. The patient, caregiver, and clinician agree upon one primary goal (Figure 2) and two secondary goals (Figure 3), which are classified into goal categories and subcategories using the Tool. All goals are set using the SMART goal setting algorithm. The goal categories are mapped based on the WHO ICF (International Classification of Functioning, Disability and Health) domains [11,12]. One goal parameter is chosen for measuring the expected changes and outcomes for each goal.

The GAS-light scoring system is used to set the baseline GAS (Goal Attainment Scale) score for the primary goal. This process is repeated for the secondary goals, resulting in a baseline GAS T-score for the group of goals for the treatment cycle about to be initiated. GAS-light, a simplified version of GAS that is designed for use in clinical practice [10].

Table 1 provides a summary of the case study goals (numbered 1 to 3), goal categories and subcategories, expected treatment benefits, goal-related spasticity patterns, ICF domains, goal parameter options to assess goal achievement, and suggested goal-related target muscles for BoNT-A injection. 

#### 2.1.1. Goal 1: To Reduce Shoulder Pain with Passive Mobilization

Goal 1 from the case study is categorized as pain. The goal is to reduce shoulder pain with passive mobilization from 8/10 to 2–3/10 (to improve sleep and the ease with which ADLs are performed). The recommended follow-up appointment is at 2–4 weeks after the BoNT-A injection. If, during the follow-up appointment, a score of −1 is found using the GAS-light, this would indicate pain >4/10 and <8/10. Before making any changes to the care plan, the clinician should investigate why the outcomes were suboptimal, taking factors such as target muscle(s) choice, toxin dosing, injection technique (guidance method), adherence to adjunctive therapy, and whether or not the goal was realistic into consideration.

#### 2.1.2. Goal 2: To Decrease Difficulty for Caregiver in Performing Hygiene to Palm of Right Hand

The goal-related spastic pattern is “clenched fist”, which makes it difficult for the caregiver to perform hand hygiene on the patient. A suggested approach is early intervention with stretching and positioning of the fingers (including splinting) alongside BoNT-A injection, whilst simultaneously engaging the spasticity multidisciplinary team. Early mobilization and splinting are important to prevent soft tissue rearrangements and shortening as a result of immobility.

#### 2.1.3. Goal 3: To Reduce Difficulty in Placing Hand in Splint by Themselves

**Figure 4:**
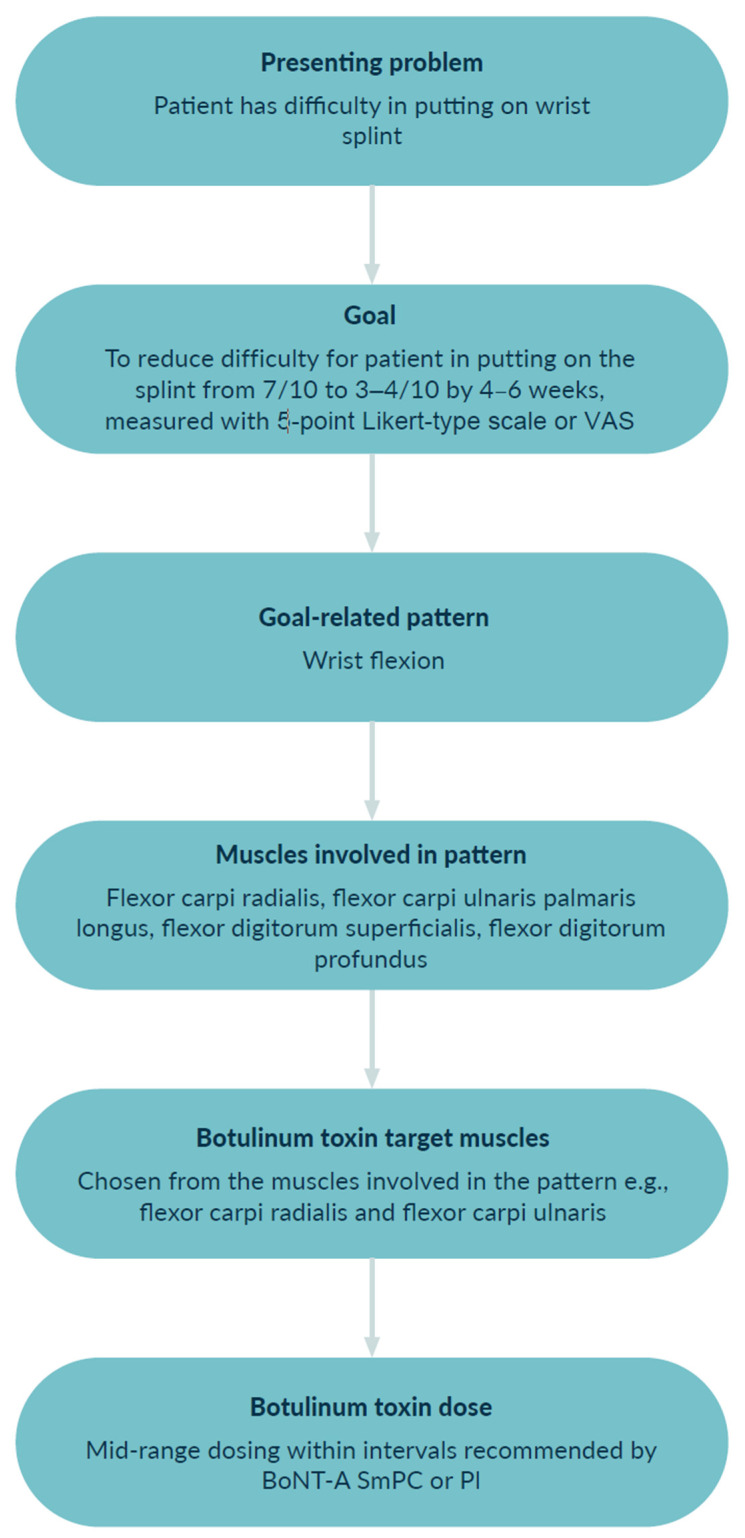
Selecting target muscles for BoNT-A injection to treat Goal 3 from case study. BoNT-A, botulinum toxin A; PI, Prescribing Information; SmPC, Summary of Product Characteristics; VAS, visual analogue scale.

The process of target muscle selection for BoNT-A injection is outlined in Figure 4 using Goal 3 from the case study. The goal is to reduce the difficulty experienced by the patient in placing the hand splint, and the goal-related spastic pattern is wrist flexion. According to a higher probability of involvement and the low severity of increased muscle tone, only two of all possibly involved muscles, for the goal-related pattern, are chosen for injection with BoNT-A. A mid-range dosing per muscle (as documented within the Summary of Patient Characteristics for the chosen formulation of BoNT-A) is used. 

A follow-up appointment is arranged at a pre-determined timepoint according to the time when goals are expected to be achieved. During follow-up, the Tool can be used to evaluate goal achievement. If the goals are not achieved as expected or if patients’ priorities change, the clinician can consider different options to optimize outcomes in future treatments. The patient, caregiver, and clinician can discuss if changes to goals are needed for the next treatment cycle. 

## 3. Discussion

### 3.1. Selection of SMART, Patient-Centred Treatment Goals 

Before commencing treatment, patient-centred treatment goals should be agreed upon between the patient, caregiver, and clinician; clearly stated and recorded at baseline; and be reiterated or adjusted and evaluated from one treatment cycle to the next [12,13]. 

Awareness of the patient’s individual needs and expectations is important in helping them to achieve their therapeutic goals [14]. The management of spasticity should be goal-focused and centred on the patient’s priority goals for treatment [1,8,15]. By setting and measuring patient-identified goals, patients are provided with psychological engagement and motivation, which empowers them in their recovery programme [8,9,16,17]. 

**Figure 5:**
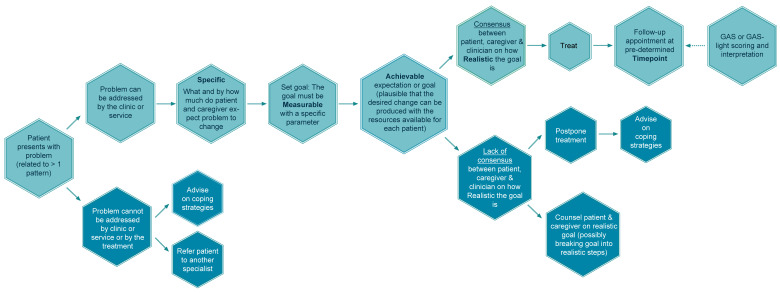
Algorithm for setting SMART treatment goals.

The Toxnet group encourages setting SMART treatment goals to ensure the patient and caregiver understand the treatment plan and what changes are expected. A SMART goal is defined as specific, measurable, attainable, realistic, and timed [12,18]. The patient and their caregiver are guided by the clinician to identify their treatment goals [15]. The goal statement should relate to the changes desired by patients regarding their condition and its impact on their life after a negotiation that ensures the expectations are realistic within one BoNT-A treatment cycle. When setting goals, the desired goals are negotiated based on the patient’s current situation, the situation they would like to progress to, and the clinician’s estimation of the realistic probability of that achievement [1,12] with the available resources and within the timeframe of one or several treatment cycles. A patient can have more than one treatment goal, and each goal should be taken through the steps of the SMART treatment goal algorithm separately (Figure 5).

Any changes or improvements must be defined as a range and stated as a variation of the chosen goal parameter from the baseline score to another score within a predefined range [12]. This helps maintain focus for patients and treating teams and provides the clinician with a clear and documented set of criteria to evaluate treatment progress against. 

According to history and physical assessment, the patient and/or caregiver and clinician agree upon one primary goal and up to three secondary goals [16,19], which are classified into goal categories and subcategories [13]. The Toxnet group suggests mapping goal categories based on the World Health Organisation (WHO) International Classification of Functioning, Disability and Health (ICF) classification [11,18]. Categorizing goals against such a common framework can be useful in understanding the impact of a condition on a patient’s life, the goals set by the patient, and the improvement obtained with the treatment [20,21,22].

One goal parameter must be chosen per goal to measure the change or outcome on an ongoing basis (e.g., numeric rating scale [NRS], visual analogue scale [VAS]), and the target time frame for achieving the desired outcome is defined for each goal. A mix of short-term and long-term goals can be set to keep the patient engaged with their individualized management programme. Realistic short-term goals can be used as a step-by-step strategy to keep the patient motivated and adherent to the long-term spasticity management programme; linking goals to one another may help the patient to see the connection between their own longer-term aspirations and any shorter-term goals suggested by the clinician [15]. 

The clinician should consider whether the patient and caregiver(s) have the coping strategies, motivation, and confidence to allow for the plan to be implemented [23,24]. There must be consensus between the patient, caregiver, and clinicians on how realistic and worthwhile each goal is, so that all parties involved are committed to work towards a common end [25]. If there is consensus, a decision is made to treat, and the Goal Attainment Scale (GAS) is used to score each goal and provide the baseline GAS score.

GAS is a patient-centred outcome measure tool for assessing the degree to which a patient’s individual treatment goals have been attained [26]. It offers a structured approach to setting goals and measuring goal achievement [27], while maintaining a patient-centred approach [18]. GAS takes into account that most patients have more than one goal and that goals vary in importance to the individual patient and their caregiver, as well as in difficulty in achieving each goal [1,12,28]. 

In GAS, ratings are prospectively and individually agreed on and based on the patient’s current and expected levels of performance or benefit, being scored relative to the expected level of achievement [19]. The Toxnet group recommends using GAS-light, a simplified version of GAS that is designed for use in clinical practice [10]; it uses a verbal descriptor rating scale, converted into numbers [12,18], as illustrated in Table 1. GAS-light considers all goals to have the same weight when calculating the GAS T-score at any moment (baseline or outcome evaluation points) [18]. 

### 3.2. Goal-Related Target Muscles and Dose Determinants

BoNT-A is a valuable tool in the multi-modal treatment approach to adult spasticity [29]. A single muscle is rarely treated in isolation; the spastic pattern and the spastic muscles responsible for it must be clearly understood by the evaluating clinician to ensure appropriate muscle targeting for BoNT-A injection. The GAS can be used to guide clinicians in deciding which muscles are a priority for injection and ensure the muscles selected are related to the goal, helping to keep the decision goal-focused and patient-focused [30].

Treatment success is also dependent on the experience and ability of the injector, both to identify and to appropriately treat the problematic muscles [1]. The individual patient’s symptoms and limitations should be considered when deciding on the target muscles for injection, the BoNT dose (per session, per muscle, and/or per injection site), the interval between treatments, and the number of target sites [31]. BoNT-A doses must be tailored to the individual patient based on their condition, the goals of treatment, the number of target muscles, and the degree of muscle hyperactivity [32].

BoNT-A is available from several manufacturers; potency units are specific to each product, and doses are not interchangeable between preparations from different manufactures. Whilst suggested BoNT-A doses are not specified in the GO-FAST Tool, the Summary of Product Characteristics (SmPC) or Product Information (PI) documents should be referred to for the minimum effective and maximum or ceiling recommended doses of each preparation per muscle and per treatment session. 

Some patients will present with both upper and lower-limb spasticity. To avoid exceeding the BoNT-A maximum recommended dose, the Toxnet group recommends a careful selection of muscles for injection according to a higher priority of involvement in the symptoms or limitations to be improved, whilst aiming to successfully meet the patient’s expectations, as defined during the goal-setting phase.

### 3.3. Follow-Up, Scoring, and Interpretation

Adequate and timely assessment is required to keep the multidisciplinary team updated on the effectiveness of the treatment approach and to facilitate its adjustment as needed. A follow-up evaluation time-point should be set for each goal a priori. The time-point will depend on the several aspects mentioned above. 

Where BoNT-A forms part of the treatment plan, its effects should be monitored over time, and standardized assessment and evaluation should be performed at realistic intervals, bearing in mind that the duration of treatment effect with BoNT-A varies between individuals [1,31]. Patients treated with BoNT-A are usually followed up at 4–6 weeks to determine the extent to which the treatment goals have been achieved, to identify any adverse effects, and to assess their compliance with the post-injection regime [1].

At the follow-up appointment, the GAS-light scoring is repeated to assess the degree to which each goal was achieved in relation to the expected outcome. The process of scoring goals has two timepoints: baseline and follow-up (Table 2).

At baseline, by definition, if the patient has a symptom or limitation that could be worse, the GAS-light score is converted to −1. In contrast, if they could not be any worse, the baseline score is −2. For example, if the goal category is pain and this is rated with an NRS at 10/10, the baseline score is −2.

At follow-up, if the goal is achieved as expected (goal-parameter change was within the predefined expected range), the rater should note 0. If the result is a little more than expected, the score is +1, and if much more than expected, the score is +2. Otherwise, if the goal has been partially achieved, the rating is −1 (if the baseline score for that goal was −1). If there was no change from the baseline, the goal achievement is rated −1 or −2, or if the follow-up situation is worse than at the baseline, the score is −2 [33].

The GAS-light results are interpreted by the clinician according to patient and caregiver advice and assessment, which determine the extent to which the goals were or were not achieved (Table 2). If a goal was not achieved as expected, then it is possible that it was unrealistic, dosing per muscle or number of muscles targeted were insufficient, injection technique or adjunctive therapy modalities were inadequate, or patient or caregiver adherence was suboptimal.

If a goal was overachieved, then it may have been set too low, and it can be amended to achieve further improvement. If it was achieved as expected and the patient is satisfied, then it could be repeated in the next treatment cycle. However, alternative goals may be set if the patient is no longer interested in the original goal.

Based on the evaluation of goal achievement (adequate magnitude of change and duration of benefit), the patient, caregiver, and clinician decide if the goal or the treatment should be maintained or altered for the next treatment cycle. 

### 3.4. The Multimodal Approach to Treatment

Spasticity management should comprise multimodal treatment delivered as multiple generalized and focal interventions as part of a patient-centric and goal-specific rehabilitation program [1]. A multimodal treatment approach can help to optimize outcomes in spasticity management by combining treatment modalities that target different aspects of the symptoms and limitations experienced by patients.

The starting point of spasticity management includes conservative measures, physical therapy and splinting, and pharmacological intervention [10]. The foundation of treatment is physical management, which is aimed at alleviating aggravating factors, relieving symptoms, improving function, and preventing deterioration [1]. Pharmacological intervention may follow; oral agents including baclofen, dantrolene, and tizanidine may be used to treat generalized spasticity [1], taking into account unwanted side effects [1]. Injectable neurolytic medications, including BoNT A and phenol, are options for focal and multifocal spasticity [1]. It is recommended that BoNT-A be used in parallel with appropriate physical or occupational therapy, postural management programs, and/or other anti-spastic strategies to meet the rehabilitation goals of the patient, caregiver, and treatment team at each treatment cycle [1].

Depending on the selected goal, there is evidence to consider adding therapeutic interventions after BoNT-A treatment to improve outcomes such as ergometer cycling, electrical stimulation, stretching, casting, taping, segmental muscle vibration, physiotherapy, extracorporeal shock wave therapy, and task-specific motor training [34,35,36,37].

Along with the choice of treatment modality, treatment outcomes may be affected by patient characteristics, clinician factors, and timing of the interventions. Factors including the patient’s history of spasticity, the rheological properties of the muscle(s), the presence of maladaptive neuroplastic changes, and sensory deficits may affect the outcomes of treatment. Whether or not the patient practices and engages in prescribed exercises or routines following a neuromuscular block with BoNT-A injection should also be considered, particularly in the presence of associated cognitive deficits and mood disturbances, as failure to do so may result in suboptimal improvement in functionality or symptoms. 

Treatment outcomes may also be influenced by the clinician’s choice of BoNT-A dilution [38] or injection guidance technique (e.g., anatomical landmark-based manual needle placement, ultrasound guidance, electromyographic, or electrical stimulation guidance) [1,39]. The clinician’s approach to goal setting, the target muscle(s) they select for injection, and the type and intensity of additional treatment modalities they choose are also key.

The timing of the intervention is an important aspect of the multimodal approach to treatment. When looking at strategies to enhance treatment outcomes, the time-sensitivity, as well as acute and delayed phases, of the complex multifactorial pathophysiology of spastic paresis must be considered.

The multimodal treatment approach considers all available treatment modalities and the timing of sequential and simultaneous treatment options in order to achieve patients’ treatment goals. Goals should be revised at the end of each treatment cycle and when they have not been achieved. The clinical team should consider whether the outcomes can be optimized with the addition of further treatment modalities or if there is a need to adjust the goals for realistic attainability. The combination of treatment modalities for each treatment cycle should be aligned with the goals defined and adapted whenever goals are changed from one cycle to the next. Through a biological synergism effect, a multimodal treatment strategy could improve the effectiveness of the individual treatments, ultimately improving patient outcomes. 

## 4. Conclusions

In conclusion, the GO-FAST Tool has been developed to help clinicians with patient-centred goal-setting and target muscle selection for BoNT-A treatment for post-stroke spasticity. It also provides support with the follow-up evaluation of goal attainment and calculation of treatment success. The utility of the Tool is demonstrated in a hypothetical case study for a primary goal (see Figure 2) and two secondary goals (see Figure 3). The Tool is freely available for rehabilitation clinicians to use from https://go-fast.toxnet.net, accessed on 27 November 2023. 

## 5. Materials and Methods

### The GO-FAST Tool

The GO-FAST Tool is designed to provide structured, coordinated support for health care professionals working in rehabilitation medicine practice to implement a patient-centred, goal-based approach to spasticity management based on outcome measurements that are practical to use in everyday clinical practice. It is intended to be used by clinicians during consultations with patients affected by spasticity. It also aims to promote standardization for data collection, potentiating real-world multicentre data pooling for research purposes.

The tool is provided as a web-based application that can be used on all tablets, smart phones, and laptops. It provides step-by-step guidance with the selection of commonly used spasticity treatment goals, associated patterns and muscles, and monitoring outcome measures to assess treatment success. Drop-down lists guide the user through the selection of goal category, sub-category, benefit, goal parameters, treatment patterns, and associated muscles to inject.

The GAS baseline score for the goal and each parameter is recorded, and the target is selected based on each parameter. The tool allows for the selection of up to five goals for each session, and a record of the process is stored on the user’s selected device. During the follow-up consultation, the user can reupload the record to document outcomes (GAS and parameter measures) for each goal, and the GAS T-score is calculated. 

## Figures and Tables

**Table 1:** Case study goal categories, expected treatment benefits, ICF domains, goal-related parameters, and suggested muscles for BoNT-A injection.

Goal No.	Goal Level	Goal Description and Desired Degree of Change	Goal Category	Goal Sub-Category	Benefit from Achieving Goal	Spasticity Pattern Related to Goal	ICF Domain	Goal Parameters to Assess Goal Achievement	Muscles Involved in Spasticity Pattern	Suggested Target Muscles for BoNT-A Injection
1	Primary	To reduce shoulder pain with passive mobilisation: from 8/10 to 4–5/10 by 2–4 weeks	Pain and symptoms	Pain	Easier to endure passive mobilization	Adduction plus internal rotation	Body Function and Structure	NRS or VAS	Pectoralis major Pectoralis minor Subscapularis Teres Major Latissimus dorsi	Pectoralis major Teres major
2	Secondary	To decrease difficulty for caregiver in performing hygiene to palm of left hand: from impossible (10/10 or 5/5) to reasonably easy (4–5/10 or 3/5) on a NRS or Likert-type scale by 4–6 weeks	Passive function	Hygiene	Easier to perform hand hygiene and reduce risk of skin problems, unpleasant odour, and social avoidance	Clenched fist	Activities and Participation	Difficulty in Performing Task: NRS 0–10 or 5-point Likert-type scale ^a^	Flexor digitorum superficialis Flexor digitorum profundus Adductor pollicis Flexor pollicis longus Flexor pollicis brevis Opponens pollicis Dorsal interossei	Flexor digitorum superficialis Flexor digitorum profundus
3	Secondary	To reduce difficulty in placing hand in splint by themselves: from 7/10 to 3–4/10 on NRS, by 4–6 weeks	Passive function	Splinting	Easier to place orthosis, hence reducing risk of deformities/contractures	Wrist flexion	Activities and Participation	Difficulty in Performing Task: VAS or NRS 0–10 or 5-point Likert type scale *	Flexor carpi ulnaris Flexor carpi radialis Palmaris longus Flexor digitorum superficialis Flexor digitorum profundus	Flexor carpi ulnaris Flexor carpi radialis

* Suggested points on 5-point Likert-type scale: impossible, very difficult, reasonably easy, easy, very easy. ADLs, activities of daily living; FIM, Functional Independence Measure; ICF, International Classification of Functioning, Disability and Health; NRS, Numeric Rating Scale; VAS, visual analogue scale; WHO, World Health Organisation.

**Table 2:** Scoring the goal using the GAS-light system. Adapted from [10].

		Verbal Rating	Numerical Conversion
**At baseline**	With respect to this goal, does the patient have?	Some function	−1
		No function (as bad as they could be)	−2
**At follow-up** Was the goal achieved?	Yes	A lot more	+2
		A little more	+1
		As expected	0
	No	Partially achieved	−1
		No change	−1 or −2
		Got worse	−2
